# Nogo Stimuli Do Not Receive More Attentional Suppression or Response Inhibition than Neutral Stimuli: Evidence from the N2pc, P_D_, and N2 Components in a Spatial Cueing Paradigm

**DOI:** 10.3389/fpsyg.2016.00630

**Published:** 2016-05-02

**Authors:** Caroline Barras, Dirk Kerzel

**Affiliations:** Faculté de Psychologie et des Sciences de l’Education, Université de GenèveGeneva, Switzerland

**Keywords:** attentional capture, attentional selection, attentional suppression, nogo, N2pc, P_D_, N2

## Abstract

It has been claimed that stimuli sharing the color of the nogo-target are suppressed because of the strong incentive to not process the nogo-target, but we failed to replicate this finding. Participants searched for a color singleton in the target display and indicated its shape when it was in the go color. If the color singleton in the target display was in the nogo color, they had to withhold the response. The target display was preceded by a cue display that also contained a color singleton (the cue). The cue was either in the color of the go or nogo target, or it was in an unrelated, neutral color. With cues in the go color, reaction times were shorter when the cue appeared at the same location as the target compared to when it appeared at a different location. Also, electrophysiological recordings showed that an index of attentional selection, the N2pc, was elicited by go cues. Surprisingly, we failed to replicate cueing costs for cues in the nogo color that were originally reported by Anderson and Folk (2012). Consistently, we also failed to find an electrophysiological index of attentional suppression (the P_D_) for cues in the nogo color. Further, fronto-central event-related potentials to the cue display showed the same negativity for nogo and neutral stimuli relative to go stimuli, which is at odds with response inhibition and conflict monitoring accounts of the Nogo-N2. Thus, the modified cueing paradigm employed here provides little evidence that features associated with nogo-targets are suppressed at the level of attention or response selection. Rather, nogo-stimuli are efficiently ignored and attention is focused on features that require a response.

## Introduction

In natural scenes, many objects compete for access to limited processing resources (Desimone and Duncan, 1995). The priority of objects for attentional selection and further processing is jointly determined by their local feature contrast (saliency) and relevance for the current tasks (Treisman and Gelade, 1980; Itti and Koch, 2001; Munoz and Fecteau, 2002; Bisley and Goldberg, 2006; Theeuwes, 2010). Traditionally, research has focused on the control of attentional selection, and bottom–up and top–down approaches have been discussed (e.g., Theeuwes, 1991; Folk et al., 1992; Fellrath et al., 2014). Less research has been devoted to the question of how distracting information is discarded. Recently, however, an electrophysiological marker of distractor suppression has been discovered (Hickey et al., 2009). The P_D_ is a greater positivity at posterior electrode sites contra–lateral to the suppressed distractor. While distractors in previous studies were stimuli with response-irrelevant features that had to be entirely ignored, we investigated the potential suppression of stimuli that required inhibition of a response. The main hypothesis was that features associated with nogo targets provide a particularly strong incentive for attentional suppression. Therefore, we expected a larger P_D_ for distractors associated with nogo stimuli than for completely response-irrelevant, neutral distractors. To foreshadow the results, we were unable to replicate previous studies reporting behavioral evidence for the suppression of distractors sharing features with nogo targets relative to neutral distractors. Accordingly, the event-related potentials (ERPs) were not different between nogo and neutral distractors, either.

### Attentional Capture, Disengagement, and Inhibition in the Spatial Cueing Paradigm

Our study uses the modified cueing paradigm developed by Folk et al. (1992) who showed that salient, but irrelevant distractors only capture attention when they share features with the target stimulus (but see Yeh and Liao, 2008). Participants searched for a color singleton in one block of trials and for a singleton onset in another block of trials. Color and onset targets were preceded by color or onset cues that were response-irrelevant and could be ignored. Shorter reaction times (RTs) with cues appearing at the target location (cueing benefits) occurred only when color targets were preceded by color cues or when onset targets were preceded by onset cues, suggesting that salient distractors capture attention only when they match the attentional set induced by the task. More recently, it has been shown that attentional capture is not exclusively determined by the match of cue and target features, but rather by the match of the perceptual relation between cue or target and the remaining display elements (Becker, 2010; Becker et al., 2013; Schönhammer et al., 2016).

However, it has been argued that the absence of congruency effects with non-matching cues does not rule out that attentional capture occurred. Theeuwes et al. (2000) argued that attention was similarly captured by matching and non-matching cues, but that attention was more rapidly disengaged when the cue did not match the target stimulus. In other words, capture of attention also occurred with non-matching cues, but may not have affected RTs because attention had already been disengaged from the cue when the target display appeared.

Two studies have addressed the hypothesis of rapid disengagement with electrophysiological measures of attention, but have concluded that attention was never captured by non-matching cues. As a measure of attentional selection, the N2pc was used. The N2pc occurs about 200–300 ms post-stimulus and is a negativity contra-lateral to the attended stimulus (Luck and Hillyard, 1994; Eimer, 1996). Some authors refer to the N2pc as Posterior-Contralateral-Negativity (PCN Töllner et al., 2012; Rangelov et al., 2013; Gokce et al., 2014). While measuring ERPs, Eimer and Kiss (2008) asked participants to search for a singleton in the target display. Matching or non-matching singletons preceded the target in the cue display. They observed that color cues resulted in an N2pc only when participants searched for color targets, but not when they searched for non-matching onset or size targets (see also Lien et al., 2008).

Further, it has been claimed that attentional disengagement is faster with cue colors that are associated with nogo-targets than with neutral cue colors. A neutral cue color refers to colors that are not response-relevant. In contrast, the nogo color is response-relevant because it tells participants to withhold the response. Belopolsky et al. (2010), participants were free to respond to either an onset or a color target (i.e., free choice of the go-target), but had to withhold the response to the alternative target (the nogo-target). For instance, if they decided to respond to color targets, they would have to refrain from responding to onset targets. A typical RT advantage of congruent over incongruent positions occurred for cues sharing the go target feature (short: go cues), but the opposite effect occurred for cues sharing the nogo target feature (short: nogo cues). Contrary to go cues, responses with nogo cues were slower when the target appeared at the cued location (cueing cost). Belopolsky et al. (2010) concluded that attention had been captured by go and nogo cues alike because of their bottom-up saliency, but was more rapidly disengaged from nogo cues than from go cues. Disengagement from nogo cues was faster because of the incentive to not process the nogo target. After very rapid disengagement, there was time to initiate suppression of the previously attended location before the target display appeared, resulting in cueing costs.

The idea that attentional capture by nogo cues was followed by rapid disengagement and suppression was challenged by Anderson and Folk (2012) who claimed that suppression may occur without previous attentional capture. In their variant of the modified spatial cueing paradigm, participants responded to the target display when the go-color was present and withheld a response when the nogo-color was present. Cues were shown in either the go color, the nogo color, or a neutral, response-irrelevant color. Consistent with Belopolsky et al. (2010), there were cueing benefits with go cues and cueing costs (incongruent faster than congruent) with nogo cues. Cues in a neutral color did not produce congruency effects. While these results are compatible with the idea of capture, rapid disengagement and subsequent suppression, results from a task in which the go color was unpredictable were not. With unpredictable go targets, cueing costs were absent for nogo cues, while cueing benefits for go cues persisted. RTs in congruent and incongruent trials with nogo cues were indistinguishable from incongruent trials with go cues. Anderson and Folk (2012) concluded that the elevated RTs in congruent trials with nogo cues relative to congruent trials with go cues resulted from inhibition of the cued location. Additionally, the similar RTs in incongruent trials with nogo and go cues showed that there was no disengagement of attention from the nogo cue. Thus, inhibitory processes may be independent of shifts of spatial attention and inhibition cannot serve as evidence for prior capture.

### Electrophysiological Correlates of Suppression

Suppression in previous studies using nogo cues was reflected in cueing costs in RTs. In the present study, we measured ERPs that are associated with attentional selection and suppression to continuously track the time course of these processes. As outlined above, the N2pc occurs in the time range between 200 and 300 ms and reflects attentional selection of contralateral stimuli. Previous studies have shown that cues in the target color capture attention, whereas neutral cues do not (Eimer and Kiss, 2008; Lien et al., 2008). We hope to replicate this result in the present study.

**FIGURE 1:**
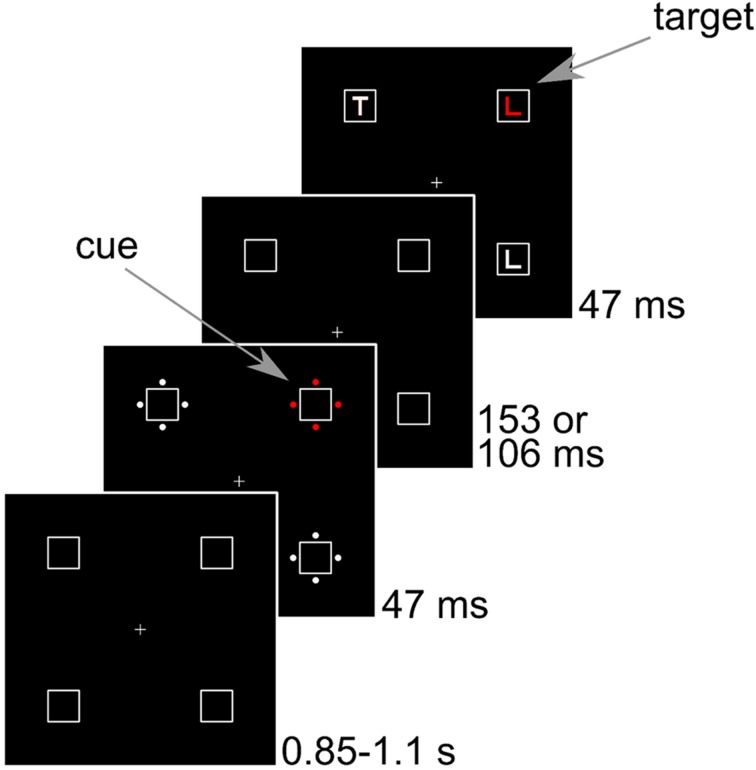
**Sequence of events in Experiments 1–3.** Stimuli are drawn to scale. The example shows a congruent trial where the cue position corresponds to the target position.

More critically, we expected a P_D_ to cues in the nogo-color. The contralateral positivity occurring in the same time range as the N2pc has been linked to distractor suppression (Hickey et al., 2009; Sawaki and Luck, 2010, 2012; Kiss et al., 2012; Burra and Kerzel, 2013; Hilimire and Corballis, 2014) and was found to be strongest for fast responses that reflect efficient distractor rejection (Jannati et al., 2013; McDonald et al., 2013; Gaspar and McDonald, 2014). While most studies have reported the P_D_ to distractors in multi-element displays, it was originally discovered to a single lateralized distractor presented simultaneously to a target on the vertical midline (Hickey et al., 2009). Hilimire et al. (2012) observed that the P_D_ was absent when only the distractor was shown and argued that the P_D_ reflects distractor suppression to facilitate processing of the target. In the modified cueing paradigm that we will employ, the cue display does not contain the target (see **Figure 1**), so there is no need for target processing. However, we think that the close temporal succession between cue and target display in the modified cueing paradigm sets off target- and distractor-related processing at the same time. Actually, the N2pc to cues in the target color (Eimer and Kiss, 2008; Lien et al., 2008) shows that the attentional set for target processing is already active when the cue display is shown.

## Experiment 1

We ran a replication of the modified cueing paradigm with nogo-stimuli by Anderson and Folk (2012) and measured ERPs. Participants responded to one color (the go-color) and refrained from responding to another color (the nogo-color) that were shown in the target display (see **Figure 1**). The irrelevant cue display preceded the target display by 200 ms. The cue was either in the response-relevant go or nogo color, or in a response-irrelevant, neutral color. Based on previous research, we predict that go cues capture attention, resulting in a cue-related N2pc. To make sure that the ERPs reflect only cue-related processing, we only analyzed cue-related ERPs until about 300 ms after cue onset (see Eimer and Kiss, 2008; Lien et al., 2008). After this time, target processing is likely to interfere with cue-related processing. Thus, we are unable to evaluate the presence of late attentional suppression occurring after the N2pc interval (Hilimire et al., 2011; Sawaki and Luck, 2012; Hilimire and Corballis, 2014).

The central question of this paper is whether there is attentional suppression of nogo cues, as indexed by the P_D_. Previous studies on this topic (Belopolsky et al., 2010; Anderson and Folk, 2012) have suggested that the incentive to not process stimuli in the nogo-color results in very fast disengagement and leaves more time for suppression. Cueing costs in RTs support this explanation and we expect to observe a P_D_ to nogo cues. Crucially, we compared nogo cues to neutral cues. If there was inhibition at the location of nogo stimuli because of the association with response inhibition, nogo cues are expected to differ from response-irrelevant, neutral cues.

A further question addressed by our experiments was whether an enhanced N2 would obtain to nogo cues and if so, whether it would be larger for nogo than for neutral cues. The N2 component of the ERP is increased at frontal sites in nogo compared to go trials (Pfefferbaum et al., 1985). The latency of the nogo-N2 (200–400 ms) may be short enough to allow for analysis of ERPs to the cue uncontaminated by target processing. The original explanation of the nogo-N2 was that it reflected response inhibition (Pfefferbaum et al., 1985; Kok, 1986; Eimer, 1993), but a number of other accounts have been proposed. The conflict monitoring account rests on the observation that the N2 was increased for rare compared to frequent events, irrespective of trial type (Nieuwenhuis et al., 2003). The response activation account claims that the N2 does not reflect inhibition in nogo-trials, but activation of the response in go-trials because the enhanced N2 persists even when participants know that they have to withhold the response on the upcoming trial, which eliminates the need for response inhibition (Bruin et al., 2001). While the N2 component is not the focus of the current paper, it is nonetheless interesting to evaluate. Previously, Eimer et al. (2009) had observed an enhanced N2 component in response to color-cues that did not match the target cues. Thus, the most straightforward prediction is that an enhanced N2 occurs for neutral and nogo cues alike because both do not match the go-color. However, based on the response inhibition account, it may be that the N2 is enhanced for nogo cues compared to neutral and go cues.

### Methods

#### Participants

Seventeen undergraduate psychology students participated for class credit, but only 13 remained in the final sample. Their mean age was 21.7 years (*SD* = 3.86). All reported normal or corrected-to-normal vision. The study was approved by the ethics committee of the Faculty of Psychology and Educational Sciences and was carried out in accordance with the Code of Ethics of the World Medical Association (Declaration of Helsinki). Informed consent was given before the experiment started.

#### Apparatus and Stimuli

Subjects were seated in a dimly lit room at 80 cm from a 21” CRT screen running at 85 Hz with a resolution of 1280 pixels × 1024 pixels. The background was black and all stimuli were matched by flicker photometry in a pilot study. The luminance of red, green, blue, and gray that resulted in minimal flicker were 9.2, 11.9, 9.3, and 11.3 cd/m^2^, respectively. The CIE coordinates (x, y) were (0.63, 0.34) for red, (0.29, 0.60) for green, (0.15, 0.07) for blue, and (0.3 0.34) for gray. In the following, eccentricities are indicated from center to center. The placeholders were squares with a side length of 1.2° that were presented at an eccentricity of 4.1° from the central fixation cross in the corners of a virtual square. In the cue display, four disks with a diameter of 0.24° surrounded each placeholder. The disks were placed at an eccentricity of 0.8° to the left, right, above, and below each placeholder. One set of disks was colored, whereas the other three sets were gray. The colored set is referred to as cue. In the target display, the letter L or T with a line length of 0.6° appeared inside each of the placeholders. There were always two Ls and two Ts in the display. The target letter was colored, whereas the other three letters were gray. Pen width was about 0.13° for the letters and 0.02° for the placeholders. The fixation cross and placeholders were visible throughout. Then, the cue display was shown for 47 ms. After an interval of 153 ms, the target display was shown for 47 ms. Thus, 200 ms elapsed between the onset of the cue and the onset of the target. We used a Latin square with three different assignments of color to cue type to counterbalance colors across participants.

#### Design

Go and nogo targets had a probability of 50%. Go, neutral and nogo cues had a probability of 33%. If cue and target displays are counted as separate events, the neutral color was shown in only 16.5% of the total number of visual events whereas the go and nogo colors were shown in 41.5%. The reason is that there were no neutral target trials. The cue was shown at the same position as the target (congruent trials) in 25% of the trials, and at a different position (incongruent trials) in 75% of the trials. All 192 combinations of the three cue colors, two target colors, four cue positions, four target positions, and two target shapes were randomly interleaved and repeated six times for a total of 1152 trials.

#### Procedure

Participants were instructed to press the left or right arrow key on a standard keyboard to indicate whether the target letter was an L or a T, but only when the target letter was in the go color. Participants responded with their right hand. When the target letter was in the nogo color, they were asked to refrain from responding. Further, they were instructed to ignore the stimuli preceding the target display and to respond as rapidly as possible, but to keep the percentage of errors below 10%. Also, they were told that the position of the cue stimulus was independent of the position of the target stimulus. Performance feedback was given after blocks of 48 trials during forced 15 s breaks. The inter-trial interval was a randomly determined interval between 0.8 and 1.3 s. The experimental session lasted about 2 h.

#### Electrophysiological Recording and Analysis

An actiCHamp amplifier (Brain Products, Gilching, Germany) with active Ag/AgCl electrodes sampled at 1000 Hz was used. We fixed 26 electrodes on the scalp, one on the outer canthi of each eye (HEOG), one above and one below the right eye (VEOG), and one on each earlobe. Cz served as online reference and AFz as ground. Oﬄine, the data were re-referenced to the average earlobes. The interval from 100 ms before to cue onset was used for baseline correction. Epochs extended from 100 ms before to 500 ms after cue onset. We excluded blinks and vertical eye movements (difference in VEOG channels exceeding ±60 mV), horizontal eye movements (difference in HEOG channels exceeding ±30 mV), and muscular artifacts (any electrode exceeding ±80 mV). We computed the average difference in the HEOG channels for left and right cues separately and rejected three participants with voltages exceeding ±3 mV. One further participant was removed because of less than 50% correct responses.

### Results: Behavior

**FIGURE 2:**
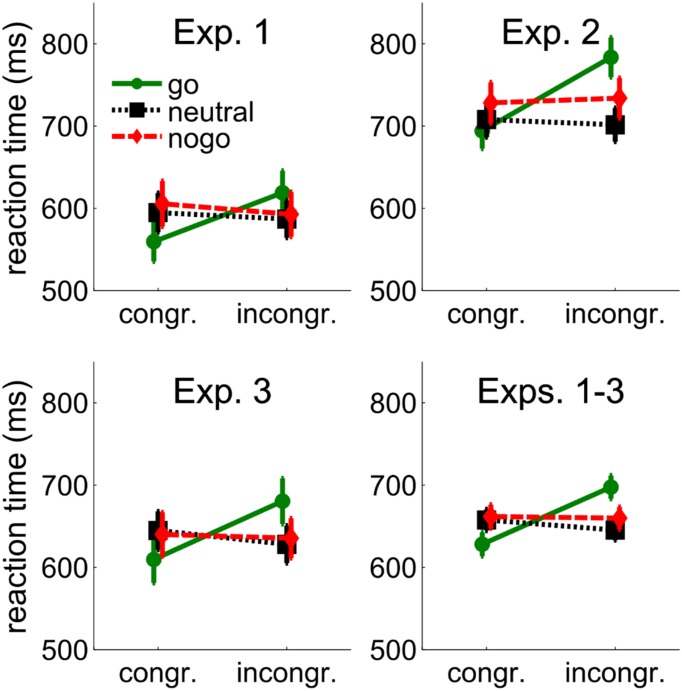
**Behavioral results from Experiments 1–3 and the average across Experiments 1–3.** Mean reaction times (RTs) are shown as a function of congruency between cue and target position, and cue color (go, neutral, and nogo). The cue color corresponded to the go- or nogo-color of the target or was different from the target colors (neutral). Error bars show between-subjects standard errors of the mean.

Trials with RTs longer than the respective condition mean plus 2.5 times the standard deviation were considered outliers. In go trials, there were 3.5% choice errors, 1.6% late trials by the online criterion of 1.5 s, and 2.3% late trials by the oﬄine outlier criterion of 2.5 standard deviations. In nogo-trials, false alarms occurred on 3.2% of the trials. Mean choice errors are shown in **Table 1** and mean RTs are shown in **Figure 2**. We subtracted performance in the spatially congruent condition from performance in the spatially incongruent condition. Positive values are referred to as cueing benefits because performance was better at the cued location. Negative values are referred to as cueing costs. Greenhouse–Geisser correction was applied when appropriate.

**Table 1:** Percentage choice errors in Experiments 1–3 as a function of cue color and spatial cue-target congruency.

	Go		Neutral		Nogo	
	Congruent %	Incongruent %	Congruent %	Incongruent %	Congruent %	Incongruent %
Experiment 1	1.4	5.5	4.4	3.0	3.1	3.6
Experiment 2	5.9	8.5	4.4	4.1	3.7	3.2
Experiment 3	3.8	4.4	3.8	4.4	3.8	4.3

#### Reaction Times

Reaction times were only available for go-targets. Individual mean RTs were entered into a 3 (cue color: go, neutral, nogo) × 2 (spatial cue-target congruency: congruent, incongruent) repeated-measures ANOVA. There was a tendency for longer RTs with nogo cues (599 ms) than with neutral (591 ms) and go cues (589 ms), *F*(2,24) = 2.84, *p* = 0.078. RTs were shorter when cue and target position were congruent (587 ms vs. 600 ms), *F*(1,12) = 14.12, *p* = 0.003. The interaction of cue color and congruency, *F*(2,24) = 49.57, *p* < 0.001, showed that the effect of spatial congruency was larger when the cue was in the go-color (59 ms) than when it was in the neutral (–8 ms) or nogo-color (–13 ms). Separate *t*-tests confirmed a cueing benefit with go cues (59 ms, 560 ms vs. 619 ms), *t*(12) = 7.81, *p* < 0.001, but cueing costs with neutral cues (–8 ms, 595 ms vs. 587 ms), *t*(12) = 2.88, *p* = 0.014, and a trend for cueing costs with nogo cues (–13 ms, 606 ms vs. 593 ms), *t*(12) = 2.15, *p* = 0.053. More importantly, however, there was no significant difference between the cueing costs with neutral and nogo cues (–8 ms vs. –13 ms), *p* = 0.407, showing that attentional suppression of cues associated with nogo stimuli was not different from attentional suppression of neutral cues.

#### Choice Errors

The same ANOVA as above was carried out on the percentage of choice errors. We confirmed a significant interaction of cue color and congruency, *F*(2,24) = 7.68, *p* = 0.003. Choice errors in go-trials were less frequent in congruent than incongruent trials (1.4% vs. 5.5%), *t*(12) = 3.05, *p* = 0.01, whereas there were no differences with neutral (3.1% vs. 3.6%), *p* = 0.528, and nogo cues (4.4% vs. 3%), *p* = 0.103.

### Results: ERPs Indicating Attentional Selection or Suppression

Based on the electrophysiological criteria enumerated above, 10% of the trials were removed. After trial rejection based on online criteria for early, late, and wrong responses and the electrophysiological criteria (see above), 86.6% of the trials remained for analysis.

The N2pc and P_D_ in response to cue display and the N2pc in response to the target display were analyzed by calculating the mean voltage in 50 ms time intervals. To determine the location of the time intervals, we first calculated the moving average (50 ms width) of the difference waveform for each condition. Then, the time window was placed on the local minimum in the interval from 200 to 300 ms for the cue-related N2pc and the local minimum in the interval from 400 to 500 ms for the target-related N2pc. We used these peaks because they were clear whereas peaks were absent or less clear in the other conditions.

**FIGURE 3:**
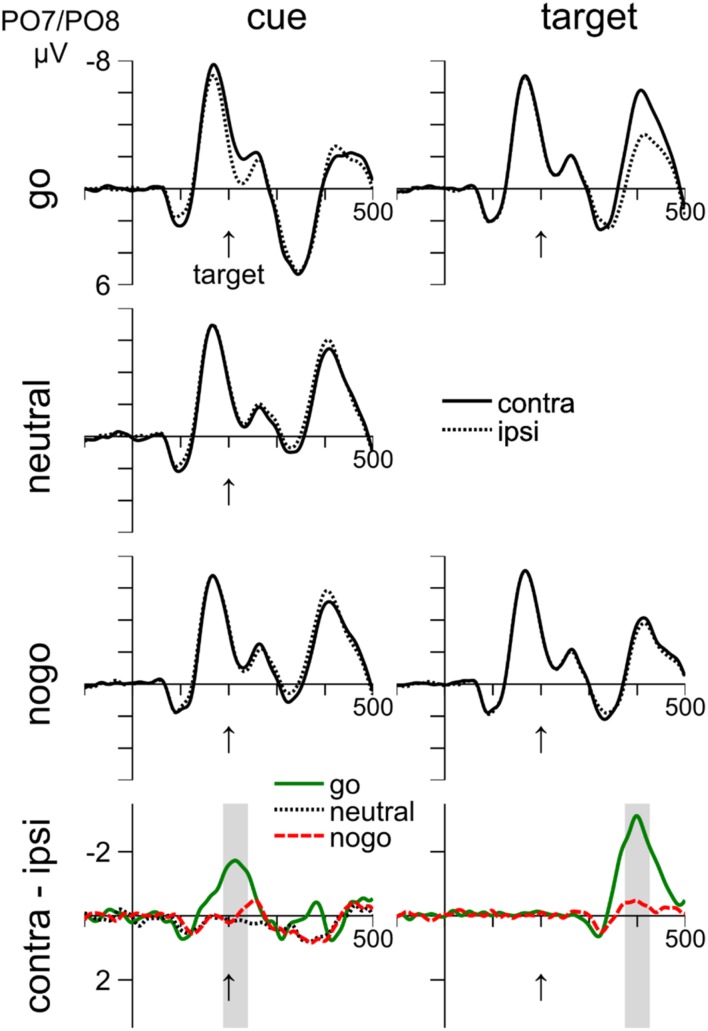
**Cue- and target-related waveforms in Experiment 1, measured at electrodes PO7/PO8.** Columns 1–2 show event-related responses to the cue and target, respectively. Rows 1–3 show responses to the go, neutral, and nogo color, respectively. Row 4 shows difference waves (contra-ipsi). On the left of the bottom row, the shaded areas show the time interval of the N2pc to the cue. On the right, the shaded area shows the time interval of the N2pc to the target. Time zero corresponds to cue onset and the vertical arrow shows the target onset at 200 ms.

**Figure 3** shows ERP waveforms measured at electrodes PO7/PO8. The left column shows the ipsi-, contralateral and difference waveforms with respect to the cue location, whereas the right column shows the waveforms with respect to the target location. Rows 1–3 show the ERPs to the go, neutral, and nogo color, respectively. Row 4 shows the difference waves. Statistics were carried out on the difference between contra and ipsilateral waveforms in the time windows shown in row 4.

#### Cue-related ERPs in the N2pc Interval

The interval from 189 to 239 ms was analyzed. The waveforms in the left column of **Figure 3** show that there was an N2pc in response to the go cue (–1.56 mV), *t*(13) = 1.56, *p* = 0.001. In contrast, there was no N2pc or P_D_ in response to the neutral cue (0.15 mV), *p* = 0.368, or to the nogo cue (0.01 mV), *p* = 0.969. The larger negativity with go cues was confirmed by a one-way ANOVA (cue color: go, neutral, nogo), *F*(2,24) = 20.82, *p* < 0.001, and by significant comparisons between go and neutral cues (–1.56 vs. 0.15 mV), *t*(12) = 4.66, *p* = 0.002, and between go and nogo cues (–1.56 mV vs. 0.01 mV), *t*(12) = 4.94, *p* < 0.001.

Inspection of **Figure 3** suggests that toward the end of the negative deflection with go cues, there was also a small N2pc to the nogo cue. We therefore placed the averaging interval on the peak of the negative deflection to the nogo cue and analyzed the interval from 222 to 272 ms. We confirmed an effect of cue color, *F*(2,24) = 9.86, *p* = 0.001, indicating that the negativity was larger for go cues (–0.93 mV) than for nogo (–0.32 mV) and neutral (0.21 mV) cues. However, the negativity was only significant for go cues, *t*(12) = 3.42, *p* = 0.005, but not for the other conditions, *p*s > 0.155.

Overall, we did not find a P_D_ to nogo cues as we had predicted. If anything, there was a small, but non-significant N2pc to nogo cues.

#### Target-related ERPs in the N2pc Interval

The waveforms in the right column of **Figure 3** show that there was an N2pc in response to the target that occurred 375–425 ms after cue onset, which was 175–225 ms after target onset. The N2pc was significant for go-targets (–2.72 mV), *t*(13) = 6.5, *p* < 0.001, and also for nogo targets (–0.4 mV), *t*(12) = 2.65, *p* = 0.021. The difference between go- and nogo-targets was significant (-2.72 mV vs. -0.4 mV), *t*(13) = 6.69, *p* < 0.001. Thus, there is clear evidence for attentional selection of go targets, whereas nogo targets were mostly ignored.

### Results: ERPs Indicating Response-Inhibition

**FIGURE 4:**
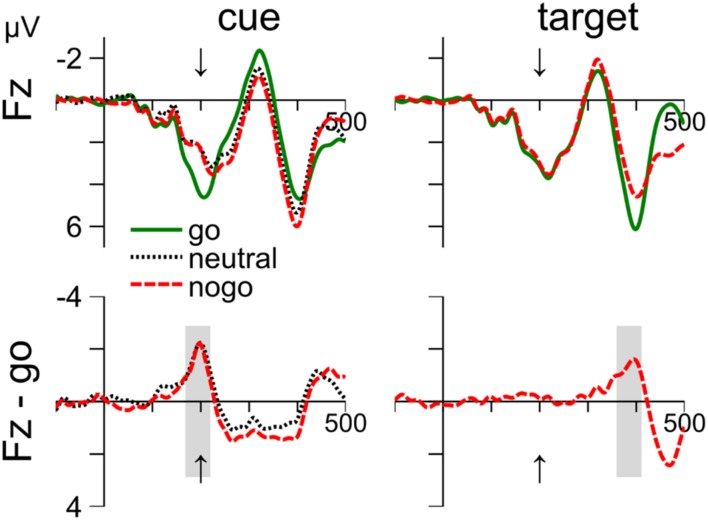
**Cue- and target-related waveforms event-related potential (ERPs) in Experiment 1, measured at electrode Fz.** Columns 1–2 show ERPs to the cue and target, respectively. Row 1 shows the mean waveform to the go, neutral and nogo color, respectively. Row 2 shows difference waves after subtraction the ERPs to go-cues **(left)** and go-targets **(right)**. The averaging windows are shown in gray. The arrow indicates time of target onset.

**Figure 4** shows that the ERPs around 200 ms after stimulus onset at electrode site Fz are remarkably similar in response to cue and target stimuli. The enhanced negativity for nogo relative to go stimuli peaked about 200 ms after stimulus onset and was followed by a positivity. The negativity correspond to the nogo-N2. The cue-related positivity extended for about 200 ms, but this may be due to the combined effects of cue- and target-related processing. In contrast, the negativity at 200 ms after cue onset coincides with target onset and can therefore be considered free of target-related processing. Further, **Figure 4** further shows that waveforms are similar for nogo and neutral cues. We used the same approach as above to search for cue- and target-related averaging windows of 50 ms between 150 and 250 ms after stimulus onset. We focus on electrode Fz, but similar results were obtained on electrode Pz. Cz was not recorded.

#### Cue-related ERPs at Fz

Waveforms are shown in the left column of **Figure 4**. The negativity was evaluated from 169 to 219 ms (see gray areas). A one-way ANOVA (cue color: go, neutral, and nogo) on the mean voltages revealed a significant main effect, *F*(2,24) = 6.46, *p* = 0.006. Follow-up *t*-test showed that the differences between nogo and go (–1.56 mV), *t*(12) = 2.37, *p* = 0.036, and between neutral and go color (-1.69 mV), *t*(12) = 3.44, *p* = 0.005, were significant. In contrast, the mean voltages with nogo and neutral cues did not differ (0.12 mV), *p* = 0.753.

#### Target-related ERPs at Fz

The waveforms are shown in the right column of **Figure 4**. Waveforms were evaluated from 360 to 410 ms. Mean voltage was more negative with nogo than with go targets (–1.3 mV), *t*(12) = 5.92, *p* < 0.001.

### Discussion

Behavioral cueing effects depended on the cue-color. Whereas a pronounced cueing benefit was observed with go cues, a trend in the opposite direction was observed with nogo cues. Importantly, the cueing effects did not differ between nogo and neutral cues, showing that the processing of nogo and neutral cues was similar. In contrast, Anderson and Folk (2012) reported significant cueing costs of about -25–40 ms with nogo cues (estimated from their **Figures 1** and **2**) that were larger than with neutral cues. It is not clear why cueing costs with nogo cues were smaller in the present study (–13 ms) and why they did not differ from neutral cues. In a related study where only go- and nogo cues were presented, cueing costs with nogo cues were of a similar size as in the present study (-9 ms in Experiment 1 of Folk and Remington, 2008).

The analysis of ERPs found an N2pc to cues in the response-relevant color, which is consistent with previous studies (Eimer and Kiss, 2008; Lien et al., 2008). However, there was little evidence for suppression of nogo cues. Analysis of the ERPs showed neither a P_D_ nor an N2pc to nogo cues and what is more, the ERPs did not differ between nogo and neutral cues. Thus, RTs and ERPs confirm that the task set results in the selection of cues that match the target color, whereas non-matching cues are neither selected nor strongly suppressed.

Analysis of the N2 confirms the conclusion that there is no differential treatment of nogo compared to neutral cues. The N2 to the cues showed an enhanced negativity for both nogo and neutral cues relative to go cues. Our findings are consistent with the idea that the nogo-N2 reflects activation of the go-response and not inhibition of the nogo response (Bruin et al., 2001), because the ERPs to go cues were different from both neutral and nogo cues, but nogo and neutral cues did not differ. Also, our results are consistent with the suggestion of Eimer et al. (2009) that the enhanced N2 reflects the top–down inhibition of features that do not match the current task set. Further, the neutral cue color was rare compared to the other colors, but the N2 was not larger for neutral cues. The conflict monitoring account would have predicted an enhanced N2 to rare events (Nieuwenhuis et al., 2003), but this was not observed.

## Experiment 2

We failed to replicate cueing costs with nogo cues in Experiment 1. However, there was a potentially important difference between Experiment 1 and the study by Anderson and Folk (2012). The SOA between cue and target display was 150 ms in Anderson and Folk (2012), but it was 200 ms in Experiment 1. We chose the longer SOA to reduce overlap between cue- and target-related ERPs. Because contingent capture depends on the time between the cue and target display (Lamy and Egeth, 2003), the change in SOA may have masked suppression of nogo cues. Therefore, we replicated Experiment 1 with a 150 ms SOA as in Anderson and Folk (2012).

### Methods

Seventeen new students from the same pool as above participated. Their mean age was 27 years (*SD* = 9.97). The stimuli were as in Experiment 1 with the exception that the interval between the cue display of 47 ms and the target display of 47 ms was reduced to 106 ms for a total presentation time of 200 ms. The number of trials was reduced to 384, as in Anderson and Folk (2012).

### Results from Experiment 2

In go-trials, there were 5% choice errors, 5.6% late trials by the online criterion of 1.5 s, and 1.8% outliers by the 2.5 SD criterion. In nogo-trials, false alarms occurred on 10.6% of the trials. Mean RTs are shown in **Figure 2**. Inspection of **Figure 2** suggests that RTs were longer in this experiment compared to the previous one. Comparison of performance between experiments is deferred to the results section of Experiment 3.

#### Reaction Times

Individual means from the go-target conditions were entered into a 3(cue color: go, neutral, and nogo) × 2 (spatial cue-target congruency: incongruent, congruent) repeated-measures ANOVA. RTs were shorter with neutral (702 ms) than with go (734 ms) and nogo cues (727 ms), *F*(2,32) = 3.98, *p* = 0.029. RTs were shorter when cue and target position were congruent (706 ms vs. 736 ms), *F*(1,16) = 16.73, *p* = 0.001, but the interaction of cue-color and congruency, *F*(2,32) = 13.83, *p* < 0.001, showed that the effect of congruency was larger when the cue was in the go-color (90 ms) than when it was in the neutral (–9 ms) or nogo-color (10 ms). Separate *t*-tests confirmed a cueing benefit with cues in the go-color (90 ms, 689 ms vs. 779 ms), *t*(16) = 5.05, *p* < 0.001. Cueing effects with neutral (–9 ms, 707 ms vs. 698 ms), *t*(12) = –0.87, *p* = 0.398, and nogo cues (10 ms, 722 ms vs. 732 ms), *t*(12) = 0.88, *p* = 0.394, failed to reach significance. As in Experiment 1, there was no significant difference between cueing effects with neutral and nogo cues (–9 vs. 10 ms), *p* = 0.268, confirming that there was no attentional suppression of cues associated with nogo stimuli beyond the attentional suppression of neutral cues.

#### Choice Errors

Choice errors are shown in **Table 1**. The same ANOVA as above on the percentage of choice errors yielded a main effect of cue color, *F*(2,32) = 9.84, *p* < 0.001. The percentage of errors was higher with go cues (7.2%) than with neutral (4.2%) and nogo cues (3.4%).

### Discussion

We confirmed the absence of cueing costs with nogo cues for an SOA of 150 ms, which contradicts the previous study of Anderson and Folk (2012).

## Experiment 3

In Experiment 1, the colors were isoluminant to avoid lateralized asymmetries in the ERPs. Even so, an early contralateral positivity, the Ppc-component (Corriveau et al., 2012; Gokce et al., 2014), was visible at about 100 ms after stimulus onset in **Figure 3**. The Ppc component is thought to reflect lateralized feature differences. We did not analyze this component, because our hypothesis concerned attentional suppression and response inhibition.

A disadvantage of using isoluminant colors is that Anderson and Folk (2012) did not use isoluminant colors. Therefore, our failure to replicate their results may be due to the discrepant luminance values. It should be noted that Anderson and Folk (2012) did not report the luminance values and changed the colors and the display device between their Experiments 1 and 2. In their Experiment 2, the RGB-values were set to a maximum for red, green, and blue, resulting in pronounced luminance differences between colors. In other words, differences in hue were associated with differences in luminance, which makes the colors more discriminable. By replicating the RGB-values in Anderson and Folk (2012), we tested whether highly discriminable colors would produce cueing costs for nogo-cues that we failed to observe in Experiments 1 and 2.

### Methods

Eighteen new students participated. Their mean age was 20.9 years (*SD* = 3.5). The methods were as in Experiment 2 with the following exceptions. The RGB-values for red, green, and blue were set to the maximum, resulting in luminance values of 16.5, 63.7, and 9.3 cd/m^2^, respectively. While we used a Latin square with three different assignments of color to cue type in Experiments 1 and 2, we counterbalanced all six possible assignments in the present experiment.

### Results

In go-trials, there were 4.2% choice errors, 2.1% late trials by the online criterion of 1.5 s, and 1.9% outliers by the 2.5 SD criterion. In nogo-trials, false alarms occurred on 5.7% of the trials.

#### Reaction Times

Reaction times are shown in **Figure 2**. Individual mean RTs from the go-target conditions were entered into a 3(cue color: go, neutral, and nogo) × 2 (spatial cue-target congruency: incongruent, congruent) repeated-measures ANOVA. RTs were shorter when cue and target position were congruent (632 ms vs. 648 ms), *F*(1,17) = 13.57, *p* = 0.002, but the interaction of cue-color and congruency, *F*(2,34) = 17.93, *p* < 0.001, showed that the effect of congruency was larger when the cue was in the go-color (71 ms) than when it was in the neutral (–17 ms) or nogo-color (–5 ms). Separate *t*-tests confirmed a cueing benefit with go cues (71 ms, 610 ms vs. 681 ms), *t*(17) = 6.01, *p* < 0.001. Cueing costs were not significant with neutral cues (–17 ms, 645 ms vs. 628 ms), *t*(17) = –1.65, *p* = 0.118, or with nogo cues (–5 ms, 640 ms vs. 635 ms), *t*(17) = –0.58, *p* = 0.571. The difference between neutral and nogo cues was not significant, *p* = 0.361.

#### Choice Errors

Choice errors are shown in **Table 1**. The same ANOVA as above did not show any significant effects, *p* > 0.33.

### Comparison between Experiments 1–3

To evaluate differences between experiments, and in particular the apparently worse performance in Experiment 2, the same ANOVA as above was performed, but we included experiment as a between-subject variable.

#### Reaction Times

A 3 (Experiments: 1, 2, and 3) × 3 (cue color: go, neutral, and nogo) × 2 (spatial cue-target congruency: incongruent, congruent) ANOVA revealed a main effect of experiment, *F*(2,49) = 6.03, *p* = 0.005, showing that RTs were longer in Experiment 2 (721 ms) than in Experiment 1 (615 ms) and Experiment 3 (640 ms). Further, it showed a tendency for an effect of cue color, *F*(2,98) = 2.78, *p* = 0.067, a significant interaction between cue color and experiment, *F*(4,98) = 2.56, *p* = 0.043, a significant effect of congruency, *F*(1,49) = 24.15, *p* < 0.001, and a tendency for an interaction between congruency and experiment, *F*(2,49) = 2.85, *p* = 0.068. Importantly, the interaction between cue color and congruency was confirmed, *F*(1.77,86.49) = 47.26, *p* < 0.001, and not modified by experiment, *p* = 0.481. Separate *t*-tests on the complete sample of 52 participants confirmed a cueing benefit with go cues (69 ms, 628 ms vs. 697 ms), *t*(51) = 7.99, *p* < 0.001. Cueing costs were significant with neutral cues (–12 ms, 657 ms vs. 645 ms), *t*(51) = –2.41, *p* = 0.019, but not with nogo cues (–2 ms, 661 ms vs. 659 ms), *p* = 0.696. Importantly, cueing costs were not significantly larger with nogo than with neutral cues (–2 ms vs. –12 ms), *t*(51) = 1.43, *p* = 0.158.

#### Choice Errors

The same ANOVA as above showed a tendency for an effect of cue color, *F*(1.67,82) = 2.96, *p* = 0.067, a significant interaction of cue color and experiment, *F*(4,98) = 3.55, *p* = 0.01, a tendency for an effect of congruency, *F*(1,49) = 3.84, *p* = 0.056, and a significant interaction of cue color and congruency, *F*(2,96) = 3.16, *p* = 0.047, that was not modulated by experiment, *p* = 0.822.

#### Effects of Age

To explain the slower RTs in Experiment 2 compared to the other experiments, we analyzed participants’ age. A one-way between-subject ANOVA found an effect of age, *F*(2,49) = 4.36, *p* = 0.018, with older participants in Experiment 2 (27 years) than in Experiment 1 (22.1 years) and Experiment 3 (20.9 years). Also, age correlated with mean RT, *r*(52) = 0.53, *p* < 0.001. Therefore, differences in age may have contributed to the overall performance drop in Experiment 2 (e.g., Zeef and Kok, 1993; Ratcliff et al., 2001). To test for this possibility, we introduced normalized age as a covariate in the above mixed-factors ANOVA. The effect of normalized age was significant, *F*(1,48) = 12.25, *p* = 0.001, while the effect of experiment was no longer significant, *F*(2,48) = 2.98, *p* = 0.06. In contrast, the crucial interaction of cue color and spatial congruency was not affected by normalized age, *p* = 0.73.

### Discussion

Similar to Experiments 1 and 2, Experiment 3 did not provide evidence for attentional suppression of nogo cues. That is, there were no cueing costs for nogo cues. The combined analysis with 52 participants confirmed the conclusion that nogo and neutral cues are not processed differently. Thus, even colors that confound hue and luminance do not replicate the pattern of results in Anderson and Folk (2012).

## General Discussion

We investigated whether cues associated with nogo-targets are more strongly suppressed than neutral cues. Previously, Belopolsky et al. (2010) and Anderson and Folk (2012) showed that nogo cues in the modified spatial cueing paradigm (Folk et al., 1992) resulted in cueing costs. Cueing costs were ascribed to inhibition of the cued location after the rapid disengagement of attention. We started from the hypothesis that cueing costs should be reflected in an electrophysiological index of attentional suppression, the P_D_. However, we were unable to find reliable cueing costs for nogo cues in three experiments and a total of 52 participants. Instead, we observed cueing benefits and an N2pc to cues in the go-color, confirming that response-relevant features capture attention (Eimer and Kiss, 2008; Lien et al., 2008). Overall, our results show that only stimuli matching the task set capture attention, whereas completely task-irrelevant, neutral cues and cues associated with response inhibition (nogo cues) neither capture attention nor result in reliable inhibition. In particular, our results do not support the hypothesis that nogo-cues entail inhibition because we failed to replicate the difference between neutral and nogo cues that was reported by Anderson and Folk (2012). The reasons for the failure are unclear, but we speculate that a sampling error produced spurious results in the previous study.

### Attentional Suppression vs. Object Updating

The present study sheds new light on the attentional strategy used to solve nogo-tasks. The hypothesis elaborated in the introduction was that participants suppress features that are associated with a nogo-stimulus at the attentional level because of the strong incentive to not respond to nogo targets. The cueing costs reported previously provided strong evidence for this idea. Our results support the alternative idea that nogo cues are mostly ignored and do not need active suppression. Nogo cues elicit neither an N2pc nor do they evoke a P_D_, while go cues elicit an N2pc. Thus, participants’ attentional set includes features requiring a response, whereas features not requiring a response are neither attended nor suppressed.

In Experiment 1, we observed small cueing costs that were not confirmed in Experiments 2 and 3. Possibly, the larger number of trials in Experiment 1 compared to Experiments 2 and 3 (1152 vs. 384) brought the cueing effects to the fore. Similarly, cueing costs with neutral cues were significant when the complete sample of 52 participants was considered. Thus, cueing costs with neutral cues are small and may not always reach significance, yielding inconsistent results. For instance, cueing costs occurred in one experiment out of two with non-singleton cues (Experiment 2 vs. 3 in Becker et al., 2013) and also with singleton cues (Experiment 1 vs. 2 in Eimer et al., 2009). In another study, cueing costs with singleton cues depended strongly on the combination of cue- and target colors (Schönhammer and Kerzel, 2013). While these reports are inconsistent, robust cueing costs have been observed in feature search with non-matching singleton cues when the cue stimuli stayed on the screen until the target appeared (Lamy et al., 2004; Carmel and Lamy, 2014, 2015). Effects of presentation time (not SOA) are unexpected from the point of view of disengagement and suppression. According to these accounts, attention would only briefly visit the location of non-matching cues.

In contrast, the object-updating account of negative cueing effects offers an explanation. In a seminal study, Kahneman et al. (1992) observed that it was easier to name a target letter that appeared in the same square as during preview compared to when it appeared in a different square. Presumably, letter and square had been integrated into an object file during preview. When the position of the target letter changed to a different square after preview, the object file had to be updated, which incurred a cost. Carmel and Lamy (2014, 2015) applied this account to the precueing paradigm: non-matching cues that appeared at the same location as the target (i.e., spatially congruent cues) resulted in the presentation of two different colors at the same position, whereas there was no change when a matching cue appeared at the target location. The change in color with congruent, non-matching cues may result in the perception of a change in the object at the cued location that requires object updating and results in cueing costs. However, the perception of a disruption in object continuity depends crucially on the presentation time of the stimuli. Carmel and Lamy (2015) observed robust cueing costs with non-matching colors when the presentation time of the cues was 150 ms, but these cueing costs disappeared with a cue presentation time of 50 ms (given a cue-target SOA of 150 ms). Presumably, the long presentation time is necessary for the creation of object files. Because presentation times in the present experiment were always short (50 ms), we suspect that the contribution of object-updating to the current results is rather small. However, the exact circumstances (stimulus size, contrast, etc.) that allow for object-file creation remain to be described in detail.

### Response Inhibition vs. Response Activation

The absence of ERP components related to the attentional suppression of stimuli favors an account of our weak cueing costs in terms of motor processes. Prinzmetal et al. (2005) showed that RT and perceptual accuracy may dissociate in cueing studies, suggesting that the effects of attention may be mediated by different mechanisms. In particular, Prinzmetal et al. (2005) argued that RTs are influenced by “channel selection,” which refers to the larger tendency to respond to cued locations compared to uncued locations. Channel selection is unrelated to enhanced perceptual processing of the cued stimuli. Nogo cues may induce the inverse tendency, that is, they may inhibit responses to the targets at the cued location while increasing the tendency to respond to targets at other locations. These processes are unlikely to have repercussions on the posterior ERP components indicating attentional selection that were the focus of the present investigation. Rather, response inhibition may be more adequately measured at frontal scalp locations. However, our results do not support the idea that response inhibition was stronger for nogo than for neutral cues, suggesting that nogo stimuli do not result in inverse “channel selection” (i.e., “channel inhibition”).

Previous ERP studies on the go/nogo paradigm have looked at the N2 and P3 components between 200 and 400 ms post-stimulus at anterior electrodes (Folstein and Van Petten, 2008). Of importance, Eimer et al. (2009) showed that the negativity at anterior electrodes was larger with non-matching than matching cues, suggesting that inhibition occurred when cues did not match the top–down set. Similarly, we observed a larger negativity for non-matching nogo cues. However, the negativity was the same for neutral cues, which suggests that the N2 does not reflect inhibition of nogo-stimuli, but rather activation of response-relevant stimuli (see Bruin et al., 2001). If the N2 reflected response inhibition, we would have expected a larger negativity to nogo cues relative to neutral and go stimuli. Thus, results from posterior and frontal electrodes support the conclusion that nogo cues do not result in more inhibition than neutral cues.

Finally, an important point is that go and nogo targets in the present study were fixed whereas they varied from trial to trial in Belopolsky et al. (2010). Belopolsky et al. (2010) observed that cueing benefits were only observed when the target on the previous and the present trial were the same. Similarly, cue-target congruency on the previous trial may affect effects of congruency on the present trial (Goller and Ansorge, 2015). As the target color was fixed during an experimental session, we cannot evaluate whether the pattern of results in the present study would change if go and nogo targets changed randomly. However, the difference between fixed and random go/nogo targets may explain why Belopolsky et al. (2010) observed stronger cueing costs than we did. The differences with respect to the study of Anderson and Folk (2012) are more difficult to explain.

In sum, we fail to replicate cueing costs to nogo cues in the modified cueing paradigm. Consistent with the lack of behavioral effects, we did not find the P_D_ component to nogo cues. In contrast, cues in the go-color evoked attentional capture at the behavioral and electrophysiological level. Our results suggest that stimuli associated with a nogo response are not suppressed at the stage of attentional selection. Rather, nogo cues are mostly ignored, which is reflected in the absence of congruency effects and electrophysiological components associated with selection and suppression.

## Author Contributions

CB and DK conceived of the experiments. CB ran the experiments and analyzed the data. CB and DK wrote the paper.

## Conflict of Interest Statement

The authors declare that the research was conducted in the absence of any commercial or financial relationships that could be construed as a potential conflict of interest.
